# HERVs: Expression Control Mechanisms and Interactions in Diseases and Human Immunodeficiency Virus Infection

**DOI:** 10.3390/genes15020192

**Published:** 2024-01-31

**Authors:** Federica Mantovani, Konstantina Kitsou, Gkikas Magiorkinis

**Affiliations:** Department of Hygiene, Epidemiology and Medical Statistics, Medical School, National and Kapodistrian University of Athens, 11527 Athens, Greece; federicam@med.uoa.gr (F.M.); konkitsou@med.uoa.gr (K.K.)

**Keywords:** HERVs, HIV, gene regulation, gene expression, silencing

## Abstract

Human endogenous retroviruses (HERVs) are the result of retroviral infections acquired millions of years ago; nowadays, they compose around 8% of human DNA. Multiple mechanisms have been employed for endogenous retroviral deactivation, rendering replication and retrotransposition defective, while some of them have been co-opted to serve host evolutionary advantages. A pleiad of mechanisms retains the delicate balance of HERV expression in modern humans. Thus, epigenetic modifications, such as DNA and histone methylation, acetylation, deamination, chromatin remodeling, and even post-transcriptional control are recruited. In this review, we aim to summarize the main HERV silencing pathways, revisit paradigms of human disease with a HERV component, and emphasize the human immunodeficiency virus (HIV) and HERV interactions during HIV infection.

## 1. Introduction

**Figure 1:**
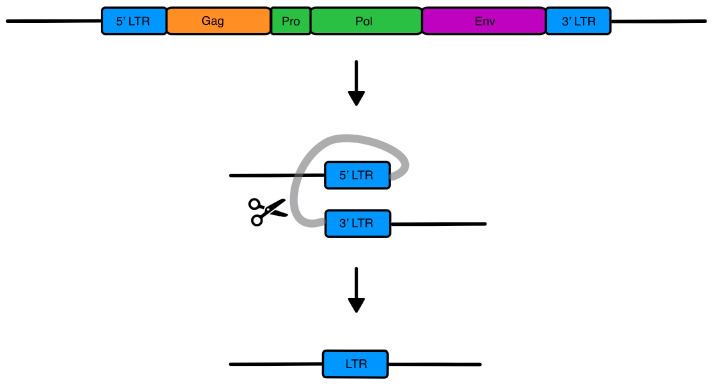
**HERV genes and solo-LTR formation**. The HERV provirus contains four main genes: Gag, encoding for the matrix, nucleocapsid, and capsid proteins; Pro, encoding for the protease; Pol, encoding the retrotranscriptase and integrase; and Env, encoding for the envelope. Flanking the genes are two LTRs with enhancer and promoter activity. Solo LTRs are created through homologous recombination, while the internal part containing the genes is lost.

Human endogenous retroviruses (HERVs) are the result of retroviral endogenization, which happened millions of years ago and currently comprise about 8% of the human genome. HERVs are classified as retrotransposons; they originally utilized reverse transcription for their duplication and reintegration in other sites of the human genome [1]. In humans, ERVs have lost their capacity for replication and retrotransposition [2], while the majority of their loci have lost the coding ability after the accumulation of point mutations, including non-sense mutations, leading to premature stop codons and frameshifts [3]. Still, though, some LTRs have retained intact ORFs with coding capacity [3]. The full-length provirus resembles the typical structure of a simple retrovirus, with the two LTRs flanking the *gag*, *pro*, *pol*, and *env* genes (reviewed in Mao et al., 2021 [4]) (Figure 1), coding for matrix, capsid, and nucleocapsid proteins (*gag*), protease (*pro*), reverse transcriptase and integrase (*pol*), and the envelope (*env*) [4,5]. HERVs have originally been classified in families, the nomenclature of which is defined by the tRNA primer binding site (PBS) at 5′ LTR of *gag* [6]; however, such categorization leads to problems regarding the actual phylogenetic relations among members of the same family [7].

Throughout evolution, certain HERV-originating proteins appear to have been co-opted by mammals to acquire new functions to the benefit of the host [8], most importantly the syncytins (HERV-W envelope: syncytin-1, HERV-FRD envelope: syncytin-2) that majorly contribute to the successful placentation [9]. Furthermore, HERV expression, namely HERV-H, HERV-L, and HERV-K [10], is higher in stem cells and during embryo development, contrary to differentiated cells, suggesting a potential role in the maintenance of the pluripotency state [10]. Additionally, full-length HERV sequences and mainly LTRs can also function as regulatory elements with promoter, enhancer, and repressor activity, poly-A signals, and alternative splicing sites in the human genome [11,12,13].

Increased HERV expression has been reported during different diseases, such as cancer and autoimmune disorders [14,15,16] and viral infections, most notably HIV-1 [17]. To date, no clear inferences have been made regarding the extent of HERV influence on modern human health and disease. The scope of this review is to summarize recent data on the physiological mechanisms of the regulation of HERV expression and their participation in the pathophysiology of selected examples of conditions, with a focus on the intra-retroviral regulation of HERVs in the presence of HIV infection and, thus, to aid the understanding of their role as disease effector factors and potential therapeutic targets.

## 2. ERV Expression Regulation Mechanisms

To maintain the physiological functions of the hosts and control HERV expression at levels that suit each cell type and developmental stage, multiple mechanisms have been developed in the course of evolutionary history. In this part of the manuscript, we aim to explore the main mechanisms that aid in the maintenance of the hosts’ homeostasis.

### 2.1. Methylation

Methylation, both regarding the retrovirally originating DNA sequences and in the frames of histone modifications, is employed for HERV transcriptional repression, with the former being mainly a characteristic of the somatic tissues, while the histone 3 lysine 9 trimethylation (H3K9me3) is mainly used as a silencing mechanism in the embryonic stem cells (ESCs) [18]. The first mechanism is used to avoid cell reprogramming, while histone methylation is more commonly associated with the prevention of unfavorable gene activation [19]. Strikingly, in the differentiating tissues, lower methylation levels and increased levels of HERV expression are found, indicating the importance of the regulation of endogenous retroviral elements in the state of pluripotency toward differentiation [10,20]. Interestingly, the type of epigenetic machinery employed appears to be dependent on the evolutionary age of the HERV insertions, as it was demonstrated that DNA methylation occurs mainly on evolutionarily younger insertions, while the histone methylation on the intermediate-age/older ones, with lower CpG density [21].

#### 2.1.1. DNA Methylation

DNA methyltransferases (DNMTs) drive cytosine methylation. In mammals, three DNTM classes (DNMT1, DNMT3A, and DNMT3B) have been extensively evaluated. Studies in mice indicate the importance of the DNMT3A and DNMT3B function in gametogenesis and early embryonic developmental stages, while low expression of these enzymes characterizes the somatic cells, rendering DNMT-mediated methylation patterns necessary for the orchestration of the epigenetic hallmarks of pluripotency and differentiation. DNMT3L, despite being a catalytically inactive homolog of DNMT3, seems to play an ancillary role in the DNMT3 machinery [22]. Regarding DNMT1, it selectively silences retrotransposon sequences in the developing mouse early embryo genomic (four- and eight-cell stage), mainly in the silencing of the LINE1 (long interspersed nuclear elements) and ERV-K sequences, while in the DNMT1-knockdown mouse embryo, enrichment of the corresponding transcripts was identified, while DNMT1 appears to be non-redundant in the process of embryogenesis [23].

#### 2.1.2. Histone Methylation

**Figure 2:**
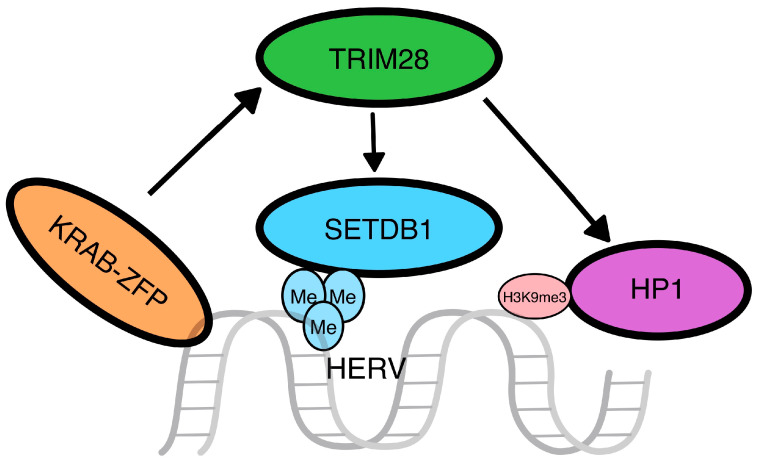
**Zinc-finger protein pathway**. The Kruppel-associated box zinc-finger proteins (KRAB-ZFP) act as recruiters for other proteins that lead to the silencing of the transposable elements. In detail, the N-term of the KRAB-ZFP recruits a tripartite motif containing 28 (TRIM28), which will work as a scaffold for the SETDB1 and HP1 proteins, a methyltransferase that will methylate the histone H3 on the lysine 9 and an H3K9me3 binding protein, respectively.

*Zinc-finger proteins (ZFPs)*: Kruppel-associated box (KRAB) zinc-finger proteins (KRAB-ZFPs) are crucial in the regulation of gene expression in mammals and are structurally characterized by two domains, the N-terminal KRAB domain and the C-terminal C2H2 zinc-finger domain, with the former functioning through the recruitment of other cellular transcription factors and the latter through binding to specific sequences for the regulation of transcription. KRAB-ZFPs appear to exert an important role in the silencing of transposable elements (TEs), including HERVs [24] and the nearby genes, through the regulation of the TE-embedded enhancers and promoters [25,26]. The KRAB domain recruits the transcription factor TRIM28 (tripartite motif containing 28 or KRAB-associated protein 1-KAP1), which functions as a scaffold for histone deacetylation proteins heterochromatin protein 1 (HP1) and histone-lysine N-methyltransferase (SETDB1) to control transcription [27], which appears to be pivotal for TE silencing in early embryos [28]. Interestingly, the co-function of TRIM28 and SETDB1 seems to enhance the transcriptional repression of HERVs in a distinct manner, more intensely than each of those factors alone [22]. KRAB-TRIM28 also regulates the expression of long-distance sequences through the extension of heterochromatin, as TRIM28 binds to the 3′ end of genes and leads to the extension of H3K9me3 and HP1β toward the 5′ end [29]. The interactions between KRAB and TRIM28 are summarized in Figure 2.

Current evidence suggests that ZFPs have been evolutionarily selected to recognize foreign sequences and lead to their silencing. This has been described in the case of ZFP809, a ZFP that appears to demonstrate a specificity in embryonic stem cells, where, despite recognizing a significant number of genomic sites, high-affinity binding and heterochromatin development takes place in primer binding sites (PBSs) associated with HERVs. ZFP809 is elevated during the state of pluripotency compared to somatic cells and, upon ZFP809 depletion in embryonic stem cells, leads to a loss of H3K9me3 and HERV transcriptional release [18]. The importance of the KRAB-ZFP system in the anti-viral protection of the host has been demonstrated in mice with ZFP961, a PBS-Lys binding protein that restrains both endogenous and exogenous retroviruses. The same study identified through chromatin immunoprecipitation sequencing (ChIP-seq analysis) similar PBS-Lys binding proteins in humans, ZNF417 and ZNF587, which, aside from silencing HERVs through facilitating their methylation, appear to restrain the HIV viral infectivity by interfering with viral transcription and integration [30]. The importance of the ZFP HERV transcription control is further shown by the HERV-T and HERV-S TRIM28-mediated silencing and ZNF genes, which appear to participate in innate immunity regulation. They were found to retain their methylation-inducing function in adult human peripheral blood mononuclear cells, where they control interferon responses [31].

Upregulation of the transcription of ZFP genes because of increased HERV element expression in tumors indicates a two-way circuit between HERV transcription and ZFP gene activation. Increased HERV and ZFP expression in these cells was linked to the better prognostic features of these cells, including behavior modifications regarding reduced growth, migrating potential, and invasiveness [32]. Despite the importance of ZFPs and the TRIM28-SETDB1 network in HERV transcriptional control, SETDB1 and H3K9me3 seem to be equally important and independently functioning in the epigenetic silencing of HERVs in differentiated cells [18].

The human silencing hub (HUSH) complex: the HUSH complex is another mechanism that is employed for the defense of the mammalian genome against “invading” sequences, mainly LINE-1 elements and HERVs, through the facilitation of histone H3 lysine 9 trimethylation (H3K9me3) [33]. HUSH has been proposed as a “universal, cell-autonomous genome-surveillance system” and could be considered an innate immunity weapon. HUSH initially recognizes and targets long, intronless transcripts, regarding non-exon organized genome as a conserved hallmark of non-mammalian origin; thus, the transcription of these “foreign” DNA elements is necessary for the H3K9me3 through HUSH [33].

**Figure 3:**
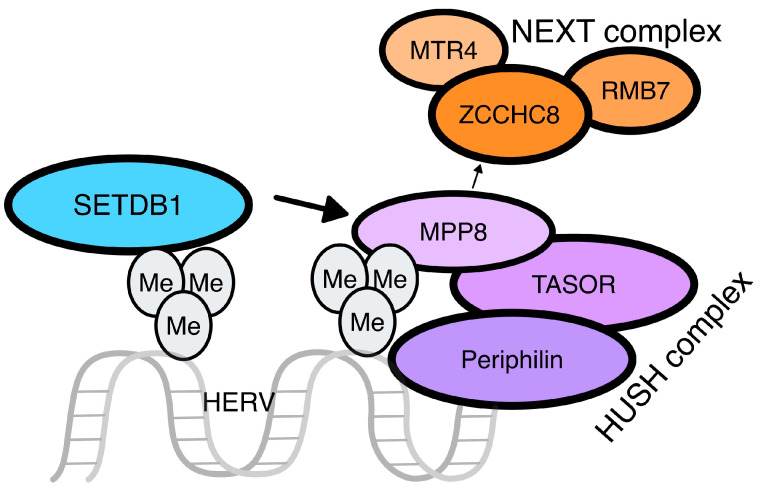
The HUSH complex. The HUSH complex is made up of three different proteins: M-phase phosphoprotein 8 (MPP8), transcription activation suppressor (TASOR), and periphilin. The latest binds on the intronless RNA molecule, while TASOR suppresses its expression. After the stop of the transcription, MPP8 interacts with ZCCHC8, a zinc-finger protein of the nuclear exosome targeting (NEXT) complex, leading to the recruitment of an RNA helicase, namely MTR4, and the RNA-binding protein RBM7. The involvement of the NEXT complex results in the decay of the HERV transcript.

This complex consists of three proteins resident in the nucleus: transcription activation suppressor (TASOR), M-phase phosphoprotein 8 (MPP8), and periphilin [34], with TASOR and periphilin existing in different isoforms as a result of gene conflict [35] (Figure 3). HUSH leads to the activation of MORC family CW-type zinc finger 2 (MORC2), an ATP-dependent chromatin remodeler that compacts chromatin, and SETDB1, for the H3K9me3 of the target sequence [36]. Studies in mice reveal the significant contribution of the HUSH complex to de novo repression of retrotransposons, especially ERVs and LINE-1, with increased specificity to evolutionarily young integrations through TRIM28 activation. Similar findings regarding HUSH silencing of at least LINE-1 elements have been described in humans [37].

Finally, these two mechanisms (DNA and histone methylation) are not separately and independently functioning but rather interact and synergize during the silencing of genomic regions, especially those of retroviral origin, for the integrity of the host, since in embryonic stem cells in mice, de novo ERV sequence methylation was dependent on the presence of KRAB-ZFPs that could recognize and could properly function on these sequences [38].

### 2.2. Histone Acetylation

Acetylation of ε-amino group of lysines is regulated by the histone/lysine acetyltransferase enzymes (HATs/KATs), leading to the more “unstable” chromatin structure and sequences more available to the transcriptional machinery, the effects of which are reversed by histone deacetylases (HDACs) [39]. Interestingly, acetylation can also be applied to non-histone proteins and also be involved in the regulation of different cellular processes, including steps of cell division and differentiation and neuronal function [40]. HDACs, similarly, can induce the deacetylation of both histone and non-histone proteins, leading to an alteration of the transcription in the first case but also to the establishment of other post-translational modifications (PTMs) on the lysines, such as methylation [39], which indicated their mediating role in other regulatory pathways described previously.

Following the consideration of HDAC inhibitors (HDACis) as a means to induce latent HIV-1 provirus expression intended for the eradication of HIV-infected cell reservoirs, interest was drawn to the potential effects of such agents on HERV expression, given their multiple implications in human disease. Results of such studies indicate a rather weak efficacy of acetylation in the control of HERV expression [41,42]. While multiple HERV loci were differentially transcribed following HDACis treatment of HIV(+) primary CD4(+) T-cells, no specific HERV expression pattern was found, such as global HERV upregulation, with the ERV-L family demonstrating a significant downregulation, and the HERV-9 LTR-12 elements were significantly enriched [41]. On the other hand, no substantially different expression of the HERV-K (HML-2) (HK2) *env* and *pol*, *syncytin-1*, and *syncytin-2* was found in another study [42].

### 2.3. Cytosine Deamination

The cytidine deaminases APOBEC3 (A3) family mediates the deamination as a mechanism of control of HERV expression which functions both on DNA and RNA molecules [43]. This system, through the expression of seven members of the A3 family, is considered a part of the human innate immunity machinery to protect the host against exogenous and endogenous retroviruses [44]. It has been demonstrated that this protein family represses retroelement expression through binding at their genomic sites in the human DNA [43], while A3G exerts its antiretroviral functions through the inhibition of the retroviral reverse transcription, aiming at the control of the retroviral replication [45].

The evolutionary expansion of the APOBEC protein family could be considered a milestone in the human genome, in terms of protection against endogenous pathogens as inferred compared to other mammals such as rodents, mainly through the A3A- and A3B-mediated regulation of LINE-1 elements and HERVs [46]. A3 genes have been amplified in the mammalian genomes in multi-species studies, and this expansion correlated positively to the germline expansion of HERVs; also, this amplification dates concurrently with ancient retroviral invasions. Finally, deamination mediated by the A3 system leads to increased A-to-G point mutation counts in ERV sequences, rendering them more susceptible to deactivation through frameshifts or premature stop codons [46].

### 2.4. Chromatin Remodeling

Chromatin remodeling, the histone shuffle to render DNA regions accessible for transcription and/or epigenetic modification, is another HERV silencing mechanism [47]. One of the mediators of such changes is the SWItch-sucrose non-fermentable (SWI-SNF) complex. Polybromo 1 (PBRM1), a member of the SWI-SNF complex, deregulation leads to the deregulated expression of HERV-ERI in clear cell renal carcinoma with a still unknown mechanism. However, repression of hypoxia inducible factor 1α (HIF-1α) seems to be a part of this SWI-SNF-mediated process [48].

Another significant protein family in the epigenetic control of HERVs is the MORC proteins that, after reducing DNA accessibility to the transcriptional machinery, aid H3K9 tri-methylation through the HUSH-complex and DNA methylation by DNMTs [49]. Interestingly, MORC3’s proper function is pivotal for ERV chromatin regulation in mice, as global ERV dysregulation was described in MORC3 knockout mice. The HUSH complex also recruits the nuclear exosome targeting (NEXT) complex as a post-transcriptional means of intronless RNA decay after its formation. HUSH and NEXT are simultaneously recruited in the nucleus, where following their binding, they regulate ERV expression both at a transcriptional and post-transcriptional level [50] (Figure 3).

## 3. HERVs in Inflammatory Diseases and Cancer

In this part of this work, we aim to revisit selected inflammatory and malignant conditions, in the pathogenesis of which HERVs have been postulated to participate.

Even though there are multiple mechanisms to circumvent the retroviral burden inflicted by HERVs on their hosts, along the long course of human–virus co-evolution, as described above, multiple diseases have been linked to their aberrant expression, with emphasis on inflammation, autoimmunity, and cancer. Strikingly, HERV dysregulated expression has been especially linked with neuro-inflammation and neuro-degeneration, with the most prominent examples of such implications being multiple sclerosis (MS) and amyotrophic lateral sclerosis (ALS). Regarding the role of HERVs in MS, HERV-W participation is highly considered a key player in the disease’s development and progression, as HERV-W concentrations in MS patients demonstrate a parallel course to the disease in terms of its relapses and remissions [51], and the presence of increased HERV-W concentrations in the cerebrospinal fluid of MS patients early in their disease indicates a worse prognosis. Additionally, strong inter-viral interactions between HERV-W and the Epstein–Barr virus (EBV) are surmised for this effect, due to the strong correlations between the activation of HERV-W in vitro and in vivo [14]. Furthermore, the implication in the immune dysregulation locally in the central nervous system (CNS) in MS can be inferred by the increase in the HERV-H and HERV-W Env epitopes on the B-cells and peripheral mononuclear cells in patients with MS relapse compared to non-relapsing patients and controls, and also, in these patients, these epitopes demonstrated increased sero-reactivity [52]. Additionally, SIRT1, an HDAC class III, has been investigated as an effector in MS, and significantly decreased SIRT1 expression both at an mRNA and a protein level was found in PBMCs during active MS phases [53]. ALS is a debilitating terminal disease of unknown etiology. Several studies have pointed to HERVs as a key mediator in the neuro-inflammation and neurodegeneration in ALS, mainly HERV-K. We show that HERV-K is activated in a subpopulation of patients with sporadic amyotrophic lateral sclerosis (ALS) and that its envelope (env) protein may contribute to neurodegeneration. Expression of HERV-K and its Env was detected in cortical and spinal neurons of ALS patients, while in animal studies, transgenic animals with the HERV-K *env* demonstrated changes in concordance with the development of motor neuron disease [54]. Additionally, HERV-K Env in neuronal extracellular vesicles has been proposed as an ALS biomarker, as their levels correlate to the disease stage and severity and ALS phenotype [55]. Finally, HERV-K was found to perpetuate the concentration of the transactive response (TAR) DNA-binding protein 43 kDa (TDP-43) in neuron cytoplasm, which is the main neural degenerative characteristic of the disease [15,56]. Simultaneous HERV-K upregulation and interferon regulatory factor 1 (IRF1), as well as nuclear factor kappa-light-chain-enhancer of activated B cells (NF-κB) isoform upregulation and nuclear translocation, has been correlated to the neurodegeneration in ALS [57].

HERVs have also been linked to the development of connective tissue diseases, mostly rheumatoid arthritis (RA) and systemic lupus erythematosus (SLE). Significantly increased levels of antibodies against an antigenic region of the HERV-K10 Gag were found in patients with RA, and it has been postulated that these antigenic regions may function as “auto-antigens” leading to the development of immune responses against the tissues they are expressed at [58]. Similar features are attributed to the transmembrane Env encoded by HERV-K in the immunological dysregulation of RA. Two HERV-K loci with intact open-reading frames for Env are expressed in neutrophils, HERV-K102 and -K108, with HERV-K102 displaying increased expression on RA neutrophils, and RA patients are producing autoantibodies recognizing the Env epitope [59]. Similar findings on HERV-K antigenicity have also been described in juvenile idiopathic arthritis [60]. Antibodies against HERVs have also been described in SLE, where expression of the envelope protein HERV-K102 and subsequent anti-HERV-K102 IgG excretion have been linked to high innate immunity activation with interferon-stimulated gene expression, similar to the effect described previously in RA [59,61]. Thus, the ERV-K102 Env activates neutrophils in SLE as well. Moreover, 124 differentially upregulated HERV loci have been correlated to SLE [62]. Interestingly, genes and small RNAs (sRNAs) encoded by HML-2 are recognized as regulatory molecules and biomarkers of diagnostic value in SLE and lupus nephritis. More specifically, five HML-2-encoded differentially expressed genes and ten HML-2-encoded sRNAs have been described that participate in the immunological processes and the progression of SLE and lupus-nephritis-mediated tissue injury, several of which have been validated through qRT-PCR in SLE patients. The most probable underlying mechanism through which SLE inflammation is mediated is the promotion of interferonogenic responses, leading to the upregulation of interferon-stimulated genes by the JAK-STAT pathway [63].

A plethora of studies have recognized correlations between HERVs and carcinogenesis. Several proteins of endogenous retroviral origin such as syncytin-1 [64] and HERV-K Rec, Env, and Np9 [65] have been proposed to have oncogenic properties [66]. Recent data point to important HERV implications in the cellular destabilization in hematological malignancies. In acute myeloid leukemia (AML), it has been seen that HERVs have a major role as enhancers, a role that has been described in other cancer types [67,68]. Chronic lymphocytic leukemia (CLL), one of the most common hematological malignancies, is divided into two subtypes, each of which has distinct molecular, as well as clinical, characteristics, most importantly affecting treatment response and overall prognosis, indolent and aggressive CLL. Recent evidence suggests certain locus-specific HERV expression profiles correlating to these forms of CLL, and strikingly, each of these differentially expressed HERV loci are associated with distinct patterns of signaling pathway modifications in the two CLL forms [69]. Regarding multiple myeloma (MM), HERV-K *env* and LTR increased expression was found as a distinctive characteristic between MM and monoclonal gammopathy of undetermined significance (MGUS) or controls, and their carcinogenic effect including, but not limited to, cell proliferation is mediated through the modification of the expression of tumor suppressive pathways, such as TP53 and CDKN1A, and the apolipoprotein B mRNA editing enzyme catalytic polypeptide-like 3F, 3G, and 3H expression [70]. Regarding solid tumors, increased HML-2 transcripts were found in neural progenitor-like (NPC-like) cells in glioblastoma cellular populations, and thus, HML-2 is important to the maintenance of stemness features. This role is surmised to be facilitated through the activation of the nuclear transcription factor octamer-binding transcription factor 4 (OCT4), which binds to the LTR5Hs of HML-2, while treatment of glioblastoma cells with antiretroviral drugs reduced reverse transcriptase activity and signified a decrease in tumor cell viability and pluripotency features [71]. Conversely, HERV-FRD-1 expression was linked to favorable outcomes in kidney renal clear-cell carcinoma patients. While lower expression of HERV-FRD-1 was found in renal clear-cell carcinoma compared to healthy tissue, its expression was linked to immunological responses against malignant cells and better disease outcomes. Further methylation studies revealed two CpG sites in HERV-FRD-1 sequences, the methylation of one of which (cg26383454) was linked to HERV-FRD-1 silencing and worse prognosis regarding disease progression and survival [72]. Alongside these oncogenic mechanisms mediated through HERVs, non-infective virions, viral-like particles, HERV-mediated oxidative stress, the promoter function of HERV LTRs, and the interplay between HERVs and exogenous oncogenic viruses have been widely discussed [16]. HERV involvement in cancer is an ongoing research subject with perspectives to be exploited as a therapeutic approach [73].

HERVs have also been implicated in the determination of the disease course and outcomes during the infection with exogenous pathogens [74]. Recently, HML-2 expression was noted as a prognostic factor of hepatitis C virus (HCV)-induced liver cirrhosis and unfavorable outcomes of HCV treatment, as increased HML-2 transcription is linked to a lack of HCV viral clearance after HCV antiviral therapy [75]. Strikingly, SARS-CoV-2 appears to modulate HERV expression, and significant global HERV upregulation has been recognized in patients with severe COVID-19, indicating their participation in the augmented inflammatory systematic process characterizing the cytokine dysregulation in COVID-19 [76]. Furthermore, HERV expression in the peripheral blood mononuclear cells (PBMCs) of COVID-19 patients can be used as a means of differentiation among stages of the infection and its severity, while distinctive patterns of HERV expression modification have been described and correspond to disease severity and clinical outcomes [77,78].

In brief, the modes of HERV participation in the diseases discussed are summarized in Table 1.

The most extensively studied example of HERVs in the outcome of infection with exogenous pathogens lies in the paradigm of HIV infection; in the next section of this review, we aim to discuss the implication of HERVs more profoundly in correlation to HIV.

## 4. HERV Expression and HIV Infection

Alterations in the cellular expression after HIV infection start taking place as soon as 30 min after the HIV’s entrance into the cell, hence before the virus integrates into the genome [79,80]. These changes imply the activation of the CD4 (cluster of differentiation 4) signaling pathway due to the binding of the virus to the receptor, leading to the interferon (IFN) and NF-κB responses, followed by the activation of stress and DNA damage responses as soon as the virus starts the integration process [79,80].

Multiple studies have identified correlations between HIV, especially in the presence of detectable viral loads, and HERV expression indicating their inter-retroviral interaction, the main HERV representative in this case being HK2 with, however, sometimes contradicting outcomes [17,81,82]. Our group has also been interested in investigating the expression of HERVs in the PBMCs of HIV patients compared to the PBMCs of healthy donors, and more specifically, we have been able to recognize generalized HERV family upregulation (including HK2), except for HERV-H, which demonstrated significant downregulation in treatment-naïve HIV patients, with similar findings in T-cell lines, namely SUP-T1 and MT4 T-cells, while we found more modest effects in cells of monocytic origin [83].

Interestingly, certain HK2 loci seem to be preferentially upregulated following HIV-1 infection; strikingly, transcriptions of the HERV-K111 and 19p13.11 proviruses have been observed only in HIV-1 patients [84]. Additionally, three HK2 proviruses, 6q25.1, 8q24.3, and 19q13.42, are upregulated in HIV-1-infected CD4(+) T-cells, whilst HK-2 12q24.33 was repressed upon HIV infection [85]. Polymorphic HERV integrations also demonstrate particular interest as a source of genetic variability in terms of HIV infection natural history; as indicated by the favorable disease course, the absence of the pericentromeric HERV-K111 integration is linked to [86].

The transactivator of transcription (Tat) is a protein of 14–16 kDa encoded by HIV-1 and used to promote the elongation of the transcript rather than the start of the transcription itself, preventing the formation of deleterious DNA products [87]. HIV-1 Tat protein can recruit HATs to change the state of the chromatin and lead to the expression of HIV once it is integrated. However, this chromatin remodeling also leads to the expression of the HK2 through the pathway of NF-κΒ and nuclear factor of activated T-cell (NF-AT) transcription factors [88]. Tat through these pathways also leads to the activation of HK2 LTR promoters. Such effects are more intense in cells that constitute HIV-1 targets, such as lymphocytes and monocytes, while HK2 gag, np9, and rec transcription are upregulated mainly in Jurkat T-cells and primary lymphocytes after HIV-1 infection [89].

Another accessory protein of HIV-1, used for the nuclear export of viral transcripts and thus increasing their cytoplasmic availability for further processing, is Rev, while a similar accessory protein, Rec, is encoded by HERV-K (type-2) [90]. This nuclear export of HERV-K (type 2) and HIV-1 is facilitated through their binding to a specific region on their mRNAs, RcRE and RRE, respectively. At least partially, the increased production of HERV-K proteins in HIV-infected patients can be attributed to the increased export of HERV-K mRNAs resulting from the structural homology between Rev and the HERV-K Rec [90]. Rev also was reported to induce HK2 expression [89,91,92].

On the other hand, retrovirally induced immune responses against HERV-K proteins have been considered to counteract HIV-1 replication and expansion, with reduced viral particle release and infectivity, and such characteristics have been attributed to HERV-K Gag [93]; at the same time, HERV Env elicits innate and adaptive immune responses [94]. Evidence of HK2 transcriptional release in HIV infection is suggested, as HK2 Env protein seems to be immunogenic, especially during HIV-1 viremia [95].

Vif (virion infectivity factor) is an additional protein encoded by HIV-1 to spread the infection in non-permissive cells. The main function of Vif, which offers a great evolutionary advantage, is that it hijacks a cellular co-transcription factor (CBF-β) to recruit a ubiquitin ligase complex that, after binding multiple members of the A3 enzyme family, namely A3D, A3F, A3G, and A3H, leads to their degradation, allowing for HIV survival and proliferation. An additional function of Vif has also been described, this time leading to the degradation of the PPP2R5 phospho-regulatory proteins, central proteins in the regulation of translation kinetics and cell cycle [96]. Since the A3 family has a wide range of non-human sequence targets, including LTR and non-LTR retrotransposons, it is reasonable to assume the results of such degradation on the expression of these elements [97]. Indeed, Vif has been implicated in the induction of HK2 immunogenic antigen expression in HIV-1-infected cells and the subsequent production of HK2-specific CD8(+) T-cells with the capacity to recognize common epitopes with HIV-1/-2 and the simian immunodeficiency virus. Vif mediates such effects, in part, in combination with other yet unknown factors, potentially through their main function with the degradation of A3 proteins. This speculation leads to the surmise that A3 proteins possibly repress HK-2 transcription, deamination-independently, by removing their transcripts as described for Alu retrotransposon transcripts and A3G [98].

Finally, viral protein R (Vpr) is an accessory protein encoded by HIV-1, together with its homologous Vpx encoded by HIV-2, and certain strains of simian immunodeficiency viruses can circumvent the anti-retroviral cellular defense mechanisms, this time through the degradation of the HUSH silencing complex, which recognizes intronless genes, leading to uncontrolled expression of integrated retroviruses, both exogenous and, consequently, endogenous as well [99].

## 5. Conclusions

In conclusion, multiple mechanisms impose the transcriptional and expressional control of HERVs to maintain the delicate equilibrium of their expression for the sake of the host’s fitness [100,101]. The increasing body of evidence leads us to contemplate HERVs as potent therapeutic targets. Concerning cancer treatment, HERV-K has been suggested as a target for chimeric antigen receptor (CAR) T-cells, with promising results in reducing cancer growth and metastatic ability [102,103]. HERVs are promising immunotherapy targets in lung adenocarcinoma, as tumor tissue exerts the formation of tertiary lymphoid structures (TLS) at its marginal locations, where local germinal center responses are triggered. Interestingly, HERV Env proteins at this location show increased antigenicity, leading to anti-tumor antibody secretion, and these effects are enhanced using immune checkpoint blockade, while the determination of the HERV expression levels in lung adenocarcinoma may be a prognostic factor of response to such therapeutic approaches [104]. Finally, recently promising results were shown for temelimab, a monoclonal immunoglobulin (Ig) G4, in the treatment for MS, targeting the HERV-W-Env, while its potential efficacy in type I diabetes mellitus has also been indicated [100,101]. Further studies will shed more light on the HERVs’ exact role in human health and disease, opening new avenues to the utilization of HERVs as potential therapeutic targets.

## Figures and Tables

**Table 1:** HERV implication in the pathogenesis and prognosis of selected examples of autoimmune conditions, malignancies, and exogenous infections (other than HIV-1).

Disease	HERV
Multiple Sclerosis	Increased HERV-W and HERV-H during disease relapses, early detection of HERV-W in cerebrospinal fluid during early stages linked to worse prognosis [51,52]
Amyotrophic Lateral Sclerosis	HERV-K Env expression correlation to disease stage, severity, phenotype, and its link to neuroinflammation pathogenesis [54,55]
Rheumatoid Arthritis	Increased levels of antibodies against HERV-K10 Gag; HERV-K102 increased expression on neutrophils and production of autoantibodies against this Env epitope [59]
Systemic Lupus Erythematosus	Anti-HERV-K102 IgG linked to innate immunity activation and interferon responses [58]
Juvenile Idiopathic Arthritis	HERV-K antigenicity [60]
Acute Myeloid Leukemia	70 HERV-K (HML-2) polymorphisms in 8q24.13 to 8q24.21 linked to disease development [68]
Chronic Lymphocytic Leukemia	Locus-specific HERV expression profiles correlating each form of the disease (indolent and aggressive); these differentially expressed HERV loci are associated with distinct patterns of signaling pathway modifications [69]
Multiple Myeloma	HERV-K *env* and LTR increased expression as a distinctive characteristic between multiple myeloma and monoclonal gammopathy of undetermined significance or controls; carcinogenic effects [70]
Glioblastoma	HERV-K HML-2 expression linked to pluripotency and stemness [71]
Kidney Renal Clear Cell Carcinoma	HERV-FRD-1 expression linked to better prognosis [72]
HCV-induced Liver Cirrhosis	HERV-K (HML-2) transcription linked to resistance to HCV clearance after antiviral therapy [75]
SARS-CoV-2	HERV-K and HERV-W as discriminative characteristics among infection stages; HERV upregulation linked to systemic inflammation [77,78]

Abbreviations: HERV (human endogenous retrovirus), Env (envelope protein), LTR (long terminal repeat), HCV (hepatitis C virus).

## Data Availability

Not applicable, no data were created in this work.
